# The Impact of Social Factors on the Duration of Hospitalization for Tuberculosis

**DOI:** 10.3390/jcm14175949

**Published:** 2025-08-22

**Authors:** Hideya Ono, Yoshiaki Minakata, Kazumi Kawabe, Seigo Sasaki, Yusuke Murakami, Takeru Sonoda

**Affiliations:** Department of Respirology, NHO Wakayama Hospital, 1138 Wada, Mihama-cho, Hidaka-gun, Wakayama 644-0044, Japan; ono.hideya.zp@mail.hosp.go.jp (H.O.); kawabe.kazumi.vn@mail.hosp.go.jp (K.K.); sasaki.seigo.su@mail.hosp.go.jp (S.S.); murakami.yusuke.am@mail.hosp.go.jp (Y.M.); sonoda.takeru.gm@mail.hosp.go.jp (T.S.)

**Keywords:** tuberculosis, duration of hospitalization, discharge destination, residence before admission, aging population

## Abstract

**Introduction:** Tuberculosis (TB) predominantly affects older adults in Japan, and prolonged hospitalization remains a challenge. This study evaluated both clinical and social factors influencing hospitalization duration. **Methods:** We retrospectively analyzed 203 patients with smear-positive pulmonary TB admitted to NHO Wakayama Hospital (2017–2022). Stepwise multiple regression was used to identify factors associated with hospitalization duration. **Results:** Key factors included time to smear negativity, duration from isolation release to discharge, independence in daily life, and discharge destination. Prolonged stays were often due to social issues, such as difficulties in arranging transfers to long-term care homes or family acceptance. **Conclusions:** While Japan is developing new discharge criteria based on clinical indicators, our findings highlight the significant impact of non-clinical, social factors on hospitalization duration. Addressing these factors is essential for effective discharge planning.

## 1. Introduction

Among infectious diseases, tuberculosis (TB) is the second leading cause of death worldwide, after COVID-19 infection. By 2022, more than 10 million new cases of TB were reported globally, and urgent responses are required [1].

The aging population is at high risk for TB [2]. The aging population (age ≥ 65 years) is expected to double to 2 billion by 2050 [3]. The Western Pacific Region (Japan, Republic of Korea, Singapore, and China) has one of the fastest growing populations of older adults globally [4,5]. In particular, Japan is the most aged country in the world (average life expectancy of 84 years) [6], with the highest proportion of older adults (30% of the total population in 2023) [7], and this proportion is expected to increase to 38.7% by 2070 [8].

In Japan, both mortality and morbidity rates of TB have been decreasing. In 1951, the mortality rate was 110.3, morbidity was 698, and TB was the leading cause of death. However, the mortality rate improved to 1.4 and morbidity to 9.2 by 2021. Since then, Japan has reached countries with a low TB incidence. Moreover, the TB demographic has shifted from primarily young individuals to elders and individuals born outside Japan [9], mirroring trends observed in other countries with a low incidence of TB [10].

These improvements can be attributed to comprehensive TB control measures initiated by the Japanese government. A complete revision of the TB Prevention Law in 1951 led to the introduction and dissemination of modern methods in the areas of chemotherapy, case detection, vaccination, surveillance, and patient management [11]. Furthermore, the Japanese version of Directly Observed Treatment, Short-course, was introduced in 2000 [12], further advancing TB control efforts.

One unique policy in Japan is to isolate patients with infectious TB in TB wards. The criteria for hospitalization in Japan encompass all sputum smear-positive patients, while discharge criteria include completion of 2 weeks of anti-TB drug therapy followed by 3 consecutive negative sputum acid-fast bacilli smears or culture tests [13]. While outpatient treatment has become predominant globally based on research conducted in Madras in the 1960s [14], strict treatment is required in Japan; in 2022, the average length of stay in TB wards was 44.5 days [15].

In recent years, the population of TB patients in Japan has aged [9], suggesting that factors influencing the duration of hospitalization may have shifted from those prevalent during periods when patients were younger. In previous reports, however, factors affecting the duration of hospitalization in Japan have primarily focused on factors affecting the duration until sputum smear negativity [16]. However, in aging societies like Japan, clinical factors alone may not sufficiently explain prolonged hospitalizations. Non-clinical elements such as discharge destination, functional independence, and difficulty in arranging post-discharge care may also play a significant role. While clinical factors—such as the time to sputum smear conversion—are well-established determinants of hospitalization length in patients with pulmonary tuberculosis, the influence of social factors remains insufficiently understood.

In this study, we examined factors influencing the duration of hospitalization, including indicators of infectivity and social factors.

## 2. Materials and Methods

### 2.1. Patients

We identified patients who were admitted to the NHO Wakayama Hospital between 1 January 2017 and 31 December 2022 and who were diagnosed with pulmonary TB based on positive sputum smear microscopy upon admission. Sputum smear microscopy was performed using auramine staining (Merck KGaA, Darmstadt, Germany). Patients who did not complete the treatment (due to death, self-discharge, or transfer due to complications), had extrapulmonary TB only, and those with negative sputum smear microscopy upon admission were excluded. This study was approved by the local ethics committee (IRB committee of NHO Wakayama Hospital; authorization number: 02-6, approval date: 17 March 2023). The contents of this study and the opportunity to reject the agreement are explained on the NHO Wakayama Hospital website.

### 2.2. Evaluation

We evaluated the factors that influenced the duration of hospitalization. We examined 27 independent variables included in the following 6 categories: demographic factors, general status, TB status, treatment factors, social status, and duration from release from isolation to discharge. The demographic factors included age, sex (male/female), and nationality (Japanese/non-Japanese). General status included history of TB treatment (yes/no), coexisting diabetes mellitus (yes/no), coexisting malignancy (yes/no), use of steroids/immunosuppressive agents (yes/no), decreased appetite (yes/no), serum albumin level, lymphocyte count, nursing care level (A/B/C), and independence in daily life (normal/J/A/B/C). TB status included drug susceptibility (no resistance/RFP resistance/INH resistance/resistance other than RFP and INH/unknown), co-existing extrapulmonary TB (yes/no), cavitation (yes/no), sputum smear microscopy at admission (±/1+/2+/3+), nucleic acid amplification test (positive/negative/unknown), detection of non-tuberculous acid-fast bacilli (yes/no), duration until sputum smear is negative, and duration until culture is negative. Mycobacterial cultures were performed in liquid media using the Mycobacteria Growth Indicator Tube (MGIT) system (Becton, Dickinson and Company, Franklin Lakes, NJ, USA), and cultures were considered negative if no growth was detected after 6 weeks of incubation. The nucleic acid amplification test was performed using the transcription–reverse transcription concerted reaction (TRC) method (Takara Bio Inc., Shiga, Japan). Treatment factors included treatment regimen (including INH and RFP/others), discontinuation of treatment due to side effects (yes/no), and change in treatment regimen (yes/no). Social status included previous residence before admission (hospital/long-term care home/home [alone]/home [a family of 2]/home [a family of >3]) and discharge destination (hospital/long-term care home/home [alone]/home [a family of 2]/home [family of >3]).

### 2.3. Definitions of Terminology

Decreased appetite was defined when the record “presence of appetite loss” was noted in the medical records. Nursing care level was classified into 3 levels according to the amount of nursing care standardized by the Ministry of Health, Labor, and Welfare Nursing System Review Committee of Japan: A—continuous observation required; B—intermittent observation (generally every 1–2 h) required; C—continuous observation not specifically required. Independence in daily life was classified into 5 levels according to the level of care needs based on criteria established by the Ministry of Health, Labor, and Welfare of Japan. N: subjects who do not have any disability and can live independently; J: subjects who have some disabilities but can live independently and go out alone; A: subjects who are generally independent in indoor activities but do not go out without assistance; B: subjects who require some assistance in indoor activities and spend most of the day in bed but can maintain a sitting position; C: subjects who spend the entire day in bed and require assistance for excretion, eating, and changing clothes. The duration From Isolation Release to Discharge was defined as the number of days from the date of release from isolation to the actual date of discharge. The criteria for permission to discharge were 3 consecutive negative sputum smear tests for patients who were discharged to home and 3 consecutive negative sputum culture tests for patients who were transferred to a hospital or a long-term care home. The isolation release date was defined as the day on which the third negative result was confirmed. Sputum tests were conducted weekly after the second week of treatment.

### 2.4. Statistical Analysis

Statistical analyses were performed using SPSS Statistics (version 29.0, IBM, Armonk, NY, USA). The normality of each factor was assessed using the Kolmogorov–Smirnov test. The correlation between the duration of hospitalization or the duration until the sputum smear was negative and each factor was assessed using Pearson’s correlation coefficient or Spearman’s correlation coefficient. Furthermore, a multiple regression analysis using the stepwise method of hospitalization duration was conducted with correlated factors. Analysis of variance was conducted with the duration of release from isolation to discharge and residence before hospitalization. When a significant difference was observed between groups, a Tukey honestly significant difference post hoc test was conducted to analyze differences within the groups.

## 3. Results

**Figure 1:**
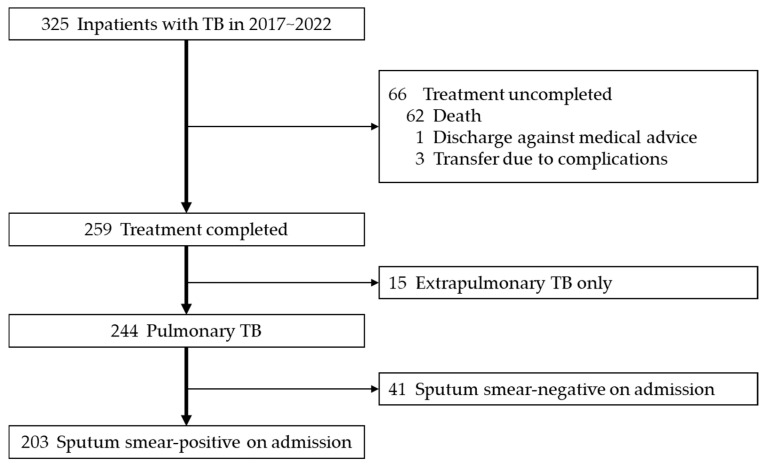
Flow diagram.

Three hundred twenty-five TB patients were admitted to our hospital between 1 January 2017 and 31 December 2022. After excluding 66 patients who did not complete treatment (62 deaths, 1 self-discharge, and 3 transfers due to complications), 15 patients with extrapulmonary TB only, and 41 patients with negative sputum smear microscopy during hospitalization, 203 patients were included in the final analysis (Figure 1).

The analyzed patients had a mean age of 73.7 ± 20.0 years, with 108 males (53.2%). A total of 158 patients had no drug resistance, 19 had INH resistance, 17 had resistance to drugs other than INH and RFP, and 9 had unknown susceptibilities. Sputum microscopy at admission showed ±1+/2+/3+ in 23/29/78/63 cases (Table 1).

The duration of hospitalization was significantly correlated with 16 factors, including age, decreased appetite, serum albumin level, lymphocyte count, nursing care level, independence in daily life, cavitation, sputum smear microscopy at admission, treatment regimen, discontinuation of treatment due to side effects, change in treatment regimen, duration until sputum smear is negative, duration until culture is negative, discharge destination, and duration from release from isolation to discharge (Table 2).

A stepwise multiple regression analysis showed that the duration of hospitalization was associated with the duration until the sputum smear was negative, duration from release from isolation to discharge, independence in daily life, and discharge destination with standardized regression coefficients of 0.68, 0.47, and 0.24, respectively, as well as F = 37.20, *p* < 0.001, and R^2^ = 0.84 (Table 3).

**Figure 2:**
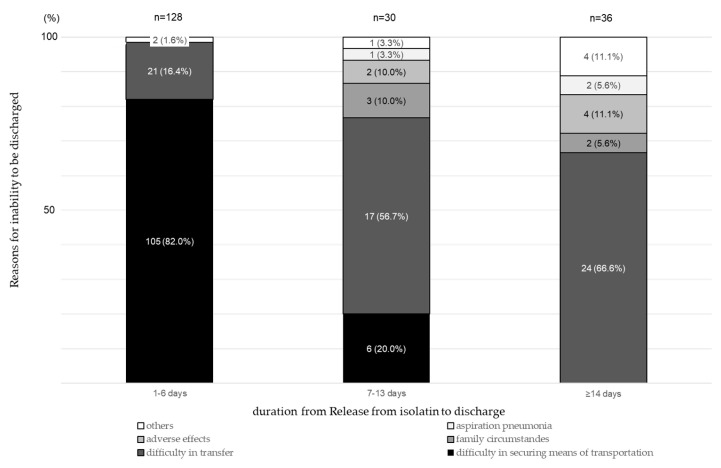
Reasons for inability to be discharged and duration from release from isolation to discharge.

Nine patients (4.4%) were discharged on the date of release from isolation. The duration from release from isolation to discharge was classified as follows: 1–6 days (*n* = 128; 63.1%), 7–13 days (*n* = 30; 14.8%), and ≥14 days (*n* = 36; 17.7%). The reasons for a duration of ≥14 days included difficulty in transferring the patient (66.6%), family circumstances (5.6%), adverse effects (11.1%), and aspiration pneumonia (5.6%) (Figure 2). The duration from release from isolation to discharge was significantly different among the previous residences before admission (F [3, 199] = 3.86, *p* < 0.01, ω2 = 0.05). A post hoc analysis showed significant differences between home (a family of >3) and hospitals/long-term care home (*p* < 0.05). It was also significantly different among discharge destinations (F [2, 200] = 14.70, *p* < 0.001, ω2 = 0.13). The post hoc analysis showed significant differences between hospitals and homes (*p* < 0.01) (Table 4).

A significant model was obtained (F = 5.23, *p* < 0.05, R2 = 3.51), and a multiple regression analysis showed that the duration until the sputum smear became negative was associated with sputum smear, cavity lesions, history of TB treatment, and discontinuation due to side effects, with standardized regression coefficients of 0.35, 0.29, 0.20, and 0.18, respectively (Table 5).

## 4. Discussion

Among patients admitted to our hospital with smear-positive pulmonary tuberculosis, the duration of hospitalization was significantly influenced by various factors. While the clinical indicator time to smear negativity showed the strongest association (β = 0.71, *p* < 0.001), several social factors—including the duration from release from isolation to discharge (β = 0.43, *p* < 0.001), level of independence in daily life (β = 0.21, *p* < 0.01), and discharge destination (β = –0.10, *p* < 0.05)—also demonstrated substantial impact, underscoring the critical role of social determinants in prolonging hospital stays.

The duration from release from isolation to discharge is one of the factors affecting the duration of hospitalization. The major obstacle to immediate discharge following release from isolation was the difficulty in securing means of transportation. However, the number of patients with difficulty in securing transportation gradually decreased over time and disappeared by day 14 after release from isolation. Among the patients hospitalized for 14 days (17.7%), the main reasons were difficulties in transfer (66.6%), family circumstances (5.6%), adverse effects of treatment (11.1%), and aspiration pneumonia (5.6%). These findings suggest that the duration from release from isolation to discharge is not merely a residual clinical period but is strongly influenced by social determinants, such as availability of post-discharge facilities, care coordination, and family-related circumstances.

In the current study, 68.0% were discharged to their homes, 24.1% were transferred to hospitals, and 7.9% to long-term care homes. The discharge destination (ω2 = 0.13) had a greater impact on the duration from release from isolation to discharge than did the pre-admission residence (ω2 = 0.05). Furthermore, post hoc comparisons revealed that the duration transferred to hospitals was significantly longer than the duration of discharge to home (*p* < 0.05) (Table 4). The possible reasons could be that accommodation beds do not become available quickly, and there are not enough staff skilled in tuberculosis care and management [17]. The shortage of care workers [18] and prejudice against tuberculosis among accepting long-term care homes [19,20] also contribute to delays in transferring patients to long-term care homes. The discharge destination was also a direct factor affecting the duration of hospitalization, as well as the duration of release from isolation to discharge.

Family circumstances were the second major reason for staying longer than 14 days, from release from isolation to discharge. This could be because of the scarcity of medical clinics capable of treating tuberculosis, increased aging, and difficulties in visiting medical clinics. Furthermore, there could be several other circumstances, including an increase in the number of families wishing to place the patients in long-term care home due to declining ADL, the shift towards nuclear families, and declining family caregiving abilities [21]. As our hospital is the only inpatient facility for tuberculosis patients in Wakayama Prefecture, patients often face long-distance travel upon discharge and feel a burden on both patients and their families. Consequently, many families prefer prolonged hospitalizations. Indeed, the duration from release from isolation to discharge for hospital transfers was 6–9 days longer than for discharge to home.

The adverse effects of treatment and aspiration pneumonia were also important reasons for a duration of >14 days from release from isolation to discharge. In Japan, the majority of tuberculosis patients are older adults, and aspiration pneumonia is frequently observed as a comorbidity during hospitalization. This clinical background likely contributes to the prolongation of hospital stays. However, to our knowledge, there are currently no published studies that systematically report the frequency or impact of aspiration pneumonia among hospitalized tuberculosis patients in Japan. Further research is warranted to clarify the role of aspiration pneumonia in the clinical course and hospitalization duration of this aging patient population. Based on our previous investigation [22], the common adverse effects leading to treatment discontinuation were hepatotoxicity (23%), skin rash (23%), biliary disorders (15%), and gastrointestinal symptoms (11%). The incidence rates of these adverse effects were consistent with those reported in the guidelines [23]. Furthermore, the median value of the onset of discontinuation was 1–4 weeks, which can significantly impact the duration from release from isolation to discharge. However, these discontinuations are unavoidable. The increasing incidence of aspiration pneumonia associated with aging could also be an unavoidable factor for the elongation from release from isolation to discharge.

Independence in daily life is a factor that affects the duration of hospitalization. Patients with lower independence in daily life tended to have longer hospital stay. A negative smear result is commonly used for discharge to home; however, negative culture confirmation is often required for transfer to hospitals or long-term care homes. Independence in daily life was significantly associated with the discharge criteria (χ^2^ [4] = 21.55, *p* < 0.001). A residual analysis of each cell indicated a significant bias: “smear-negative” was more frequent in patients with normal independence in daily life (*p* < 0.001), and “culture-negative” was more common in patients with independence in daily life at levels A (*p* < 0.05) and C (*p* < 0.01). Patients with lower independence in daily life were more frequently transferred to hospitals or long-term care homes, resulting in a greater number of patients with culture-negative discharge and a prolonged hospital stay.

The duration until sputum smear negativity was one of the major factors affecting the duration of hospitalization. Consistent with previous reports, it was associated with bacterial load, cavitary lesions, history of tuberculosis treatment, and discontinuation due to adverse effects [24,25,26]. Age could influence the duration until smear negativity in patients with a median age of 41 years [26]; however, it did not influence this duration in another study with older adults groups of 65–74 years of age and ≥75 years of age [20]. In the current study, age was not identified as a related factor. This may be due to the median age of the older adult population, which was 81 years.

The present study was associated with several limitations. This investigation was conducted at our hospital, which is located in an area experiencing aging and depopulation, and thus did not reflect the general situation in Japan, including urban areas. However, considering the nationwide population decline, this study may provide insights into future situations in Japan. The majority of subjects in this study were older adults, and the number of non-Japanese patients, which has been increasing in recent years, was very low. Different factors may influence the duration of hospitalization of non-Japanese patients. Further investigations that include non-Japanese patients are required.

## 5. Conclusions

This study identified both clinical and social factors associated with the duration of hospitalization for patients with smear-positive pulmonary tuberculosis. While the time to sputum smear conversion was the strongest determinant, the period from release from isolation to discharge also had a substantial impact, likely reflecting social barriers such as delays in discharge coordination and post-discharge care arrangements. Discharge destination and independence in daily life were also meaningful contributors to prolonged hospital stays.

To effectively reduce the length of hospitalization, discharge policies should incorporate social considerations and promote early coordination with long-term care facilities.

## Figures and Tables

**Table 1:** Patients’ characteristics.

Characteristics		No.
Age ^a^ (years)		73.7 ± 20.0, 81 (69, 88)
Sex	Male/Female	108/95
Nationality	Japan/Other countries	197/6
Tuberculosis treatment history	Yes/No	19/184
Diabetes	Yes/No	31/172
Malignancy	Yes/No	14/189
Use of corticosteroid or immunosuppressant	Yes/No	11/192
Anorexia	Yes/No	43/160
Albumin ^b^ (mg/dL)		3.31 ± 0.82
Lymphocyte count ^b^ (/μL)		1077 ± 518
Nursing level	A/B/C	13/106/84
Daily Life Independence Level	Normal/J/A/B/C	89/26/31/28/29
Drug sensitivity	no resistance/resistant to RFP/resistant to INH/other resistance/unidentified)	158/0/19/0/17/9
Extrapulmonary Tuberculosis	Yes/No	28/175
Cavitary lesion	Yes/No	66/137
Sputum smear	±/1+/2+/3+	23/39/78/63
Nucleic acid amplification method	positive/negative/not examined	26/169/8
Treatment regimen	HR contained regimen/others	171/32
Discontinuations Due to Adverse Effects	Yes/No	49/154
Change in anti-tuberculous drugs	Yes/No	47/156
Detection of non-tuberculous mycobacteria	Yes/No	33/170
Duration until negative smear ^a^ (days)		25.0 ± 18.4, 20 (14, 35.5)
Duration until negative culture ^a^ (days)		44.1 ± 24.7, 42 (21, 61)
Residence before hospitalization	Hospital/Long-term care home/Home(alone)/Home (family of 2)/Home (family of ≥3)	1/22/57/44/79
Discharge destination	Hospital/Long-term care home/Home(alone)/Home (family of 2)/Home (family of ≥3)	49/16/39/37/62
Duration from release from isolation to discharge ^a^ (days)		8.3 ± 12.6, 3 (1, 9)

^a^ Mean ± standard deviation, median (IQR); ^b^ Mean ± standard deviation.

**Table 2:** Correlation (vs. duration of hospitalization).

	Correlation (vs. Duration of Hospitalization)	
	Correlation Coefficient	*p* Value
Age	0.31	<0.01
Sex	0.02	0.82
Nationality	−0.10	0.18
Tuberculosis treatment history	0.07	0.32
Diabetes	0.31	0.51
Malignancy	0.02	0.28
Use of corticosteroid or immunosuppressant	−0.10	0.48
Anorexia	0.08	0.01
Albumin	0.31	<0.01 ^a^
Lymphocyte count	0.02	<0.01
Nursing necessity level	−0.10	0.04
Daily Life Independence Level	0.09	<0.01
Drug sensitivity	0.31	0.81
Extrapulmonary tuberculosis	0.02	0.49
Cavitary lesion	−0.10	<0.01
Sputum smear	0.10	<0.01
Nucleic acid amplification method	0.31	0.13
Treatment regimen	0.02	0.01
Discontinuation due to adverse effects	−0.10	<0.01
Change in anti-tuberculous drugs	0.11	<0.01
Detection of non-tuberculous mycobacteria	0.02	0.05
Duration until negative smear	−0.10	<0.01
Duration until negative culture	0.12	<0.01
Residence before hospitalization	0.32	0.08
Discharge destination	0.02	<0.01
Duration from release form isolation to discharge	0.47	<0.001

^a^ Pearson product moment correlation coefficient.

**Table 3:** Multiple regression analysis of duration of hospitalization.

Independent Variable	Regression Coefficient	Standardized Regression Coefficient (β)	Standard Error	t	*p* Value	VIF	95% CI
intercept	11.71		4.88	2.40	0.02		
Duration until negative smear	0.90	0.71	0.05	18.98	<0.01	1.01	[0.80, 0.99]
Duration from release form isolation to discharge	1.12	0.43	0.11	10.40	<0.01	1.20	[0.91, 0.99]
Independence in daily life	4.23	0.21	0.89	4.73	<0.01	1.40	[2.46, 6.00]
Discharge destination	−1.87	−0.10	0.88	−2.12	<0.05	1.45	[−3.62, −0.13]
R^2^	0.83						
F	4.50				<0.05		

**Table 4:** Post hoc analysis of discharge destination and the duration from release from isolation to discharge.

Discharge Destination	Discharge Destination	Mean Difference	Standard Deviation	Significance Probability	95% CI
Hospital	Long-term care home	3.21	2.69	0.75	[−4.19, 10.61]
	Home (alone)	5.95 *	2.00	0.03	[0.44, 11.46]
	Home (family of 2)	9.36 **	2.03	0.00	[3.76, 14.95]
	Home (family of ≥3)	9.01 **	1.78	0.00	[4.10, 13.92]
Long-term care home	Home (alone)	2.74	2.77	0.86	[−4.89, 10.37]
	Home (family of 2)	6.15	2.79	0.18	[−1.54, 13.83]
	Home (family of ≥3)	5.80	2.62	0.18	[−1.40, 13.00]
Home (alone)	Home (family of 2)	3.41	2.14	0.50	[−2.49, 9.30]
	Home (family of ≥3)	3.06	1.91	0.50	[−2.19, 8.31]
	Home (family of ≥3)	−0.35	1.94	1.00	[−5.68, 4.99]

* *p* < 0.05, ** *p* < 0.01.

**Table 5:** Multiple regression analysis of time to negative smear.

Independent Variable	Regression Coefficient	Standardized Regression Coefficient (β)	Standard Error	t	*p* Value	95% CI
intercept	9.20		5.97	1.54	0.13	
Sputum smear	9.06	0.35	2.00	4.52	<0.001	[5.09, 13.03]
Cavitary lesion	15.30	0.29	4.13	3.72	<0.001	[7.16, 23.50]
TB treatment history	17.59	0.20	6.70	2.62	<0.05	[4.31, 30.87]
Treatment interruption due to adverse effect	9.96	0.18	4.16	2.39	<0.05	[1.72, 18.20]
R^2^	0.35					
F	5.73				<0.05	

## Data Availability

The clinical data used to support the findings of this study are available from the corresponding author upon request.

## Abbreviations

TB	Tuberculosis
INH	Isoniazid
RFP	Rifampicin
